# Cellular mechanisms of copper neurotoxicity in human, differentiated neurons

**DOI:** 10.1007/s00204-024-03921-0

**Published:** 2024-12-16

**Authors:** Barbara Witt, Sharleen Friese, Vanessa Walther, Franziska Ebert, Julia Bornhorst, Tanja Schwerdtle

**Affiliations:** 1 Division of Food Chemistry and Toxicology, Department of Chemistry, RPTU Kaiserslautern-Landau, Erwin-Schroedinger-Str. 52, 67663 Kaiserslautern, Germany; 2 Department of Food Chemistry, Institute of Nutritional Science, University of Potsdam, Arthur-Scheunert-Allee 114-116, Nuthetal, 14558 Potsdam, Germany; 3 TraceAge – DFG Research Unit on Interactions of Essential Trace Elements in Healthy and Diseased Elderly (FOR 2558), Berlin-Potsdam-Jena-Wuppertal, Germany; 4 Food Chemistry With Focus On Toxicology, Faculty of Mathematics and Natural Sciences, University of Wuppertal, Wuppertal, Germany; 5 Max Rubner-Institut, Federal Research Institute of Nutrition and Food, Haid-Und-Neu-Straße 9, 76131 Karlsruhe, Germany

**Keywords:** Copper, Neurons, Neurotoxicity, Mitochondria, Oxidative stress

## Abstract

Copper (Cu) is an essential trace element involved in fundamental physiological processes in the human body. Even slight disturbances in the physiological Cu homeostasis are associated with the manifestation of neurodegenerative diseases. While suggesting a crucial role of Cu in the pathogenesis, the exact mechanisms of Cu neurotoxicity involved in the onset and progression of neurological diseases are far from understood. This study focuses on the molecular and cellular mechanisms of Cu-mediated neurotoxicity in human brain cells. First, the cytotoxic potential of Cu was studied in fully differentiated, human neurons (LUHMES cells). Lysosomal integrity was considerably affected following incubation with 420 µM CuSO_4_ for 48 h. Further mechanistic studies revealed mitochondria and neuronal network as most susceptible target organelles (already at 100 µM CuSO_4_, 48 h), while the generation of reactive oxygen species turned out to be a rather later consequence of Cu toxicity. Besides Cu, the homeostasis of other elements might be involved and are likely to contribute to the pathology of Cu-mediated neurological disorders. Besides Cu, also effects on the cellular levels of magnesium, calcium, iron, and manganese were observed in the neurons, presumably aggravating the consequences of Cu neurotoxicity. In conclusion, insights in the underlying mode of action will foster the development of treatment strategies against Cu-mediated neurological diseases. Particularly, the interplay of Cu with other elements might provide a powerful diagnostic tool and might be used as therapeutic approach.

## Introduction

Copper (Cu) is an important micronutrient in our food. As essential trace element, it is involved in vital physiological processes in the human body. Besides its pivotal role in Cu-dependent proteins, Cu is recently discussed to even exert a neuromodulatory role in neurons being released in the synaptic cleft (Bhattacharjee et al. 2020; Bost et al. 2016). Under physiological conditions, the Cu homeostasis is strictly regulated, since even slight imbalances can have severe consequences for the human health. Cu dyshomeostasis has been linked to certain diseases, particularly affecting the brain (e.g., Wilson disease, Alzheimer’s disease). Both, altered Cu levels and a change in Cu distribution in the brain have been associated with neurological disorders; however, the exact role of Cu in the pathogenesis still remains unclear (Bulcke et al. 2017; Manto 2014; Scheiber et al. 2014).

In general, Cu homeostasis in the brain is regulated via the barrier systems of the brain (Abbott et al. 2010; Joshi et al. 2021). Regulatory homeostatic mechanisms involve Cu transporters such as Ctr1 and ATP7A/B. Moreover, transport proteins, so-called chaperons such as ATOX1, play an important role in the cellular distribution of Cu. There are also Cu-binding proteins such as metallothioneins or glutathione (GSH) to keep free Cu at a low level within the cells (An et al. 2022; Malosio et al. 2020).

Excess Cu levels are known to impair the neuronal function, while the cellular mechanisms of Cu neurotoxicity and implications within neurological disorders are far from understood. Highest brain Cu levels are found in substantia nigra (Gromadzka et al. 2020; Joshi et al. 2021; Kawahara et al. 2021; Manto 2014). Human dopaminergic neurons in the substantia nigra are reported as major target cells of Cu neurotoxicity (Yu et al. 2008). Discussed cellular modes of action are mitochondrial impairment and induction of oxidative stress. Cu is reported to affect mitochondrial proteins and to disrupt the mitochondrial structure leading to functional impairment in cell and animal models. Particularly, brain mitochondria were highly susceptible to Cu toxicity (Borchard et al. 2018; Bulcke et al. 2017; Zischka et al. 2011). Due to its redox activity, Cu contributes to the formation of free radicals via Fenton-like redox reaction (Stohs and Bagchi 1995). In the brain, further modes of reactive oxygen species (ROS) induction are discussed: Cu promotes the oxidation of dopamine resulting in diverse reactive species. Moreover, the amyloid peptide seems to enhance oxidative stress through redox cycling of Cu in context of Alzheimer’s disease (Eskici and Axelsen 2012; Huang et al. 1999). In fact, other essential elements are reported to be involved in the pathogenesis of neurological disorders as well. Dysregulated iron (Fe) and calcium (Ca) brain levels are associated with neuronal dysfunction and neuronal loss in context of neurodegeneration (Glaser et al. 2019; Wang et al. 2020).

The aim of this study was to characterize the neurotoxic mechanisms of excess Cu in human, differentiated, dopaminergic neurons (Lund human mesencephalic (LUHMES) cells). Besides general cytotoxic effects, focus was set on the adverse impact on mitochondria and neuronal network as potential neuronal target organelles. Moreover, effects on antioxidative defense and ROS induction were studied in the neurons. Since other essential elements are discussed to contribute to pathological changes in context of neurological diseases, interferences with the homeostasis of elements including magnesium (Mg), Ca, Fe, and manganese (Mn) were analyzed in parallel. Findings of this study help to shed light on the pathological conditions induced by a Cu dyshomeostasis on cellular levels in human, dopaminergic neurons. Thus, implications in the onset and progression of Cu-related neurodegenerative diseases will be revealed, providing a fundamental basis to develop adequate therapeutic approaches and strategies.

## Materials and methods

### Chemicals

Advanced DMEM/F12 and N2-supplement were purchased from Life Technologies GmbH (Darmstadt, Germany). L-Glutamine was obtained from Biochrom (Berlin, Germany), and recombinant human basic fibroblast growth factor (FGF) and recombinant human glial cell-derived neurotrophic factor (GDNF) were supplied by R&D Systems (Wiesbaden-Nordenstadt, Germany). Copper (II) sulfate (anhydrous, > 99% purity) was purchased from Carl Roth (Karlsruhe, Germany. All other chemicals were obtained from Merck KGaA (Darmstadt, Germany) or Carl Roth (Karlsruhe, Germany).

### Cell cultivation and incubation

LUHMES cells were cultivated as described before (Lohren et al. 2015; Lotharius et al. 2002, 2005; Witt et al. 2017). In brief, flasks and plates were precoated with poly-L-ornithine and fibronectin before use. Proliferating, undifferentiated LUHMES cells were cultivated in advanced Dulbecco’s modified Eagle’s medium (DMEM)/F12 supplemented with L-glutamine, N2-supplement, and recombinant human basic fibroblast growth factor. Differentiation process was initiated by tetracycline, resulting in shutdown of v-myc expression (Lotharius et al. 2002). From then on, differentiated cells were cultivated in advanced DMEM/F12 supplemented with L-glutamine, N2-supplement and dibutyryl cyclic adenosine monophosphate sodium salt, tetracycline, recombinant human glial cell-derived neurotrophic factor. The cells reached maturation stage following 1 week cultivation. Neurons were seeded in precoated dishes at a defined density of 150,000 cells/cm^2^ for the experiments. Following treatment with copper (II) sulfate (CuSO_4_) for 48 h, the respective experiments were conducted.

### Cytotoxicity testing

#### Lysosomal integrity (Neutral red)

Lysosomal integrity was monitored via neutral red uptake. Neurons were incubated with 66.7 µg/mL neutral red (3-amin-7-dimethylamino-2-methylphenazine hydrochloride) in advanced DMEM/F12 (3 h, 37 °C). Neurons were washed with 0.5% formaldehyde/PBS and treated with 50% EtOH/1% acetic acid/PBS to solubilize the incorporated dye. Absorbance was measured using a Tecan plate reader (Abs: 540 nm, Ref: 690 nm; Tecan infinite 200 Pro, Tecan Austria GmbH, Grödig, Austria).

#### Cell number (Hoechst)

Cell number was indirectly assessed via Hoechst staining. The fluorescent bisbenzimidazole Hoechst 33258 binds to cellular DNA, and thus, cell number can be assessed via the quantification of cell nuclei. Briefly, neurons were washed with PBS and fixed with 3.7% formaldehyde/PBS (10 min, 37 °C), permeabilized with 2.2% Triton™ X-100/PBS (10 min, 37 °C), and treated with 6 µM Hoechst 33,258/PBS (30 min, 37 °C). Fluorescence was measured using a Tecan plate reader (Ex: 355 nm, Em: 460 nm).

#### Cell viability (Resazurin reduction assay)

Cell viability was measured using resazurin reduction assay. Neurons were incubated with 5 µg/mL resazurin in advanced DMEM/F12 (3 h, 37 °C). Thereafter, fluorescence was measured using a Tecan plate reader (Ex: 540 nm, Em: 590 nm).

### Mitochondria and oxidative stress

#### Mitochondrial membrane potential (MitoTracker^®^ Orange).

Mitochondrial activity was assessed via MitoTracker^®^ Orange staining as described before (Chazotte 2011; Ebert et al. 2016). In brief, neurons were stained with 300 nM MitoTracker^®^ Orange (30 min, 37 °C) and fixed with 3.7% formaldehyde/PBS (10 min, 37 °C). The fluorescence was measured using a Tecan plate reader (Ex: 544 nm, Em: 590 nm).

#### ROS (MitoSOX™ Red)

Mitochondrial ROS were measured using the dye MitoSOX™ Red. This dye is accumulated in the mitochondria and is specifically oxidized by superoxide to the fluorescent 2-hydroxyethidium. This endpoint was modified to a screening method (96-well plate format) (Witt et al. 2021). In brief, neurons were washed twice with warm PBS. 2.5 µM MitoSOX^TM^ Red/PBS was applied (30 min, 37 °C). Fluorescence was measured using a Tecan plate reader (Ex: 510 nm, Em: 580 nm). Antimycin was used as positive control (100 µM, 1 h).

#### Cellular glutathione homeostasis

Cellular glutathione (GSH) and glutathione disulfide (GSSG) levels were measured using an enzymatic recycling assay based on the conversion of 5,5’-dithiobis-(2-nitrobenzoic acid) to a chromophore as described before (Leffers et al. 2013; Witt et al. 2021). In brief, external calibration was used to quantify GSH and GSSG levels in lysed cell pellets. The conversion to the chromophore was monitored at 412 nm using a Tecan plate reader. GSH plays an important role in the antioxidative defense system of the neurons, and thus, the ratio of GSH/GSSG was used to assess the cellular redox status (Rahman et al. 2006).

### Neuronal network

#### Neuronal network (βIII-tubulin)

Effects on the neuronal network were assessed via immunostaining of the neuronal cytoskeletal protein βIII-tubulin as described before (Scholz et al. 2011; Witt et al. 2017). Neurons were fixed on precoated glass coverslips with 3.7% formaldehyde [30 min, room temperature (RT)], washed with 0.05% Tween^®^ 20/PBS, and permeabilized with 0.2% Triton™ X-100/PBS (15 min, RT). Neurons were blocked with 1% BSA/PBS (30 min, RT), and incubated with the primary antibody [mouse anti-Tubulin βIII (TUBB3), Clone: Tuj1, 1:500, overnight, 4 °C] and thereafter with the secondary antibody (Alexa Fluor^®^ 488 goat anti-mouse IgG, 1:1000, 1 h, RT). DAPI was used to stain the nuclei. Images were obtained using a fluorescence microscope (Leica DMB 6, Wetzlar, Germany, 20 × objective).

#### Western blot (βIII-tubulin)

To quantify effects on the neuronal network, protein expression of βIII-tubulin was detected via western blot analysis. In brief, cell pellets were resuspended in RIPA buffer (10 mM TRIS, 150 mM NaCl, 1 mM EDTA, 1% Triton™ X-100, 1% sodium deoxycholate, 0.1% SDS, and 1 µg/mL protease inhibitors), sonicated, and centrifuged (10,000x*g*, 20 min, 4 °C). Protein content was quantified via Bradford assay. Samples were diluted in Laemmli buffer/H_2_O to 20 µg protein/25 µL. Proteins were separated via polyacrylamide gel electrophoresis (12.5% SDS-PAGE) and transferred to a nitrocellulose membrane. After blocking the membrane (5% skimmed milk powder/TBS, 1 h, RT), the membrane was first incubated with the primary antibody (mouse anti-Tubulin β3, 1:10,000, 2 h, RT) and thereafter with the secondary antibody (anti-mouse m-IgGκ BP-HRP, 1:10,000, 1 h, RT). Clarity™ Western ECL Substrate and ChemiDOC™ MP Imaging Systems were used to visualize the protein bands (Bio-Rad Laboratories GmbH; Munich, Germany). βIII-tubulin was normalized to β-actin as housekeeping gene (primary antibody: rabbit anti-β-actin, 1:10,000, overnight, 4 °C; secondary antibody: anti-rabbit IgG, HRP linked, 1:10,000, 1.5 h, RT).

### Cellular copper levels and element homeostasis

Total Cu levels were measured in the neurons applying ICP-MS/MS to assess cellular bioavailability as described before (Lohren et al. 2015; Witt et al. 2021). To study potential interactions with other elements, cellular levels of selected elements (Mg, Ca, Mn, and Fe) were analyzed in parallel. In brief, neurons were lysed, and protein levels of lysates were measured via Bradford assay. Cell lysates were digested with nitric acid (24 h, RT) and element levels were measured using ICP-MS/MS (Agilent 8800 ICP-QQQ-MS, Agilent Technologies, Waldbronn, Germany). External calibration based on an internal standard (Ge) was used for quantification. Blank controls, quality controls, and certified reference material NIST Trace Elements in Natural Water 1640A were regularly measured for quality assurance. Cellular element levels were related either to respective protein amounts or to cell volumes for obtaining µM concentration ((7.05 ± 0.22) × 10^–13^ L).

### Statistics

All experiments were independently conducted at least three times. Mean and standard deviation (SD) were derived from raw data. Graph Pad Prism was used to perform statistical analysis via ANOVA one-way test followed by Dunnett’s test. Significant levels: * *p* < 0.05, ** *p* < 0.01, and *** *p* < 0.001.

## Results

### Cytotoxicity testing

**Fig. 1:**
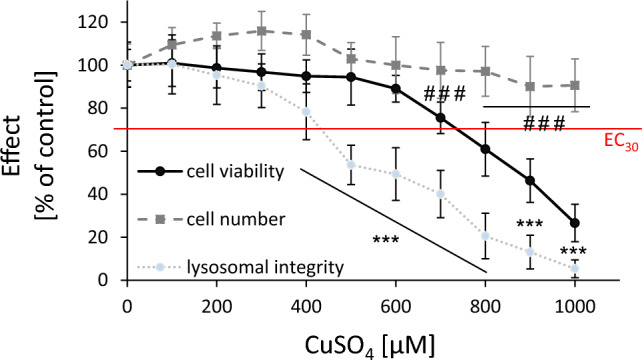
General cytotoxicity: Cell viability (Resazurin), cell number (Hoechst), and lysosomal integrity (Neutral red) were assessed in the human, differentiated neurons (LUHMES cells) following 48 h incubation with CuSO_4_. Shown are mean values of at least three independent experiments ± SD related to untreated control. EC_30_: effective concentration with 30% impact. ###/*** *p* < 0.001 (compared to untreated control) based on ANOVA one-way test followed by Dunnett’s test (color figure online)

First, the cytotoxic potential of CuSO_4_ was characterized in fully differentiated, human neurons (LUHMES cells). Following 48 h of Cu incubation, cell number, cell viability, and lysosomal integrity were concentration-dependently reduced (see Fig. 1). Interestingly, lysosomal integrity was mostly affected with an EC_30_ value (effective concentration with 30% impact) of 420 µM. The other cytotoxicity endpoints were comparatively less sensitive with EC_30_ values of approximately 720 µM (cell viability) and > 1000 µM (cell number), respectively.

In vitro studies with human brain cells are scarce in context of Cu toxicity. Recent studies with human neuroblastoma cells (SH-SY5Y) showed pronounced cytotoxic effects with an EC_50_ (50% impact) of 720 µM following 24 h Cu incubation (Pradhan et al. 2023). In another study, cytotoxic effects were already observed at 150 µM after 24 h treatment of neuroblastoma cells. In accordance with our study, cell viability also turned out to be more sensitive as compared to cell number (Arciello et al. 2005).

### Mitochondria and oxidative stress

**Fig. 2:**
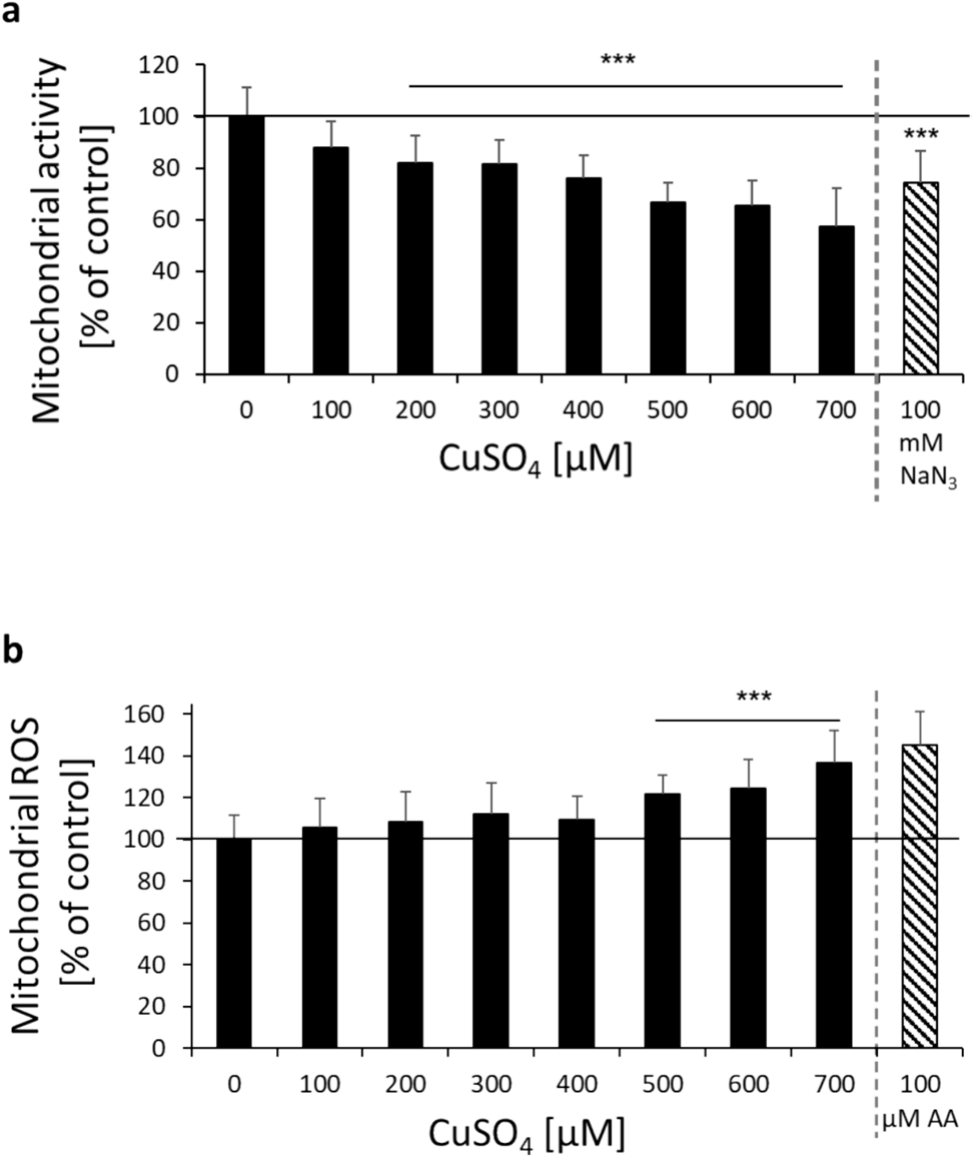
Mitochondria: **a** Mitochondrial activity was assessed applying Mitotracker^®^ Orange in human, differentiated neurons (LUHMES cells) following 48 h incubation with CuSO_4_ and the positive control NaN_3_, respectively (100 mM, 30 min). Mitotracker^®^ Orange was related to cell number, to exclude cytotoxic effects. **b** Mitochondrial ROS were assessed applying MitoSOX^TM^ Red in human, differentiated neurons (LUHMES cells) following 48 h incubation with CuSO_4_ and the positive control antimycin (AA), respectively (100 µM, 60 min). Shown are mean values of at least three independent experiments + SD. *** *p* < 0.001 (compared to untreated control) based on ANOVA one-way test followed by Dunnett’s test

The brain is a highly energy-dependent organ. Since mitochondria are supposed to be target organelles of Cu toxicity, this organ is particularly affected (Borchard et al. 2018). Consequently, brain mitochondria are discussed to play a key role in the pathogenesis of Cu-associated neurodegenerative diseases. First, the mitochondrial membrane potential was assessed in human neurons. Cu showed pronounced effects on mitochondrial activity with similar sensitivity as compared to lysosomal integrity (see Fig. 2a). Interestingly, mitochondria seem to be even more sensitive in this neuronal brain cell model as compared to others. Mitochondrial impairment has been also observed in human astrocytes, however, only at beginning cytotoxic concentrations (Witt et al. 2021).

Closely linked to an impaired mitochondrial function is the induction of ROS. Considering the high redox activity of Cu, oxidative stress might be an important mode of action in the course of Cu neurotoxicity. Mitochondrial ROS were directly analyzed in human neurons using MitoSOX™ Red. In fact, ROS were significantly induced only at cytotoxic Cu levels (see Fig. 2b).

**Fig. 3:**
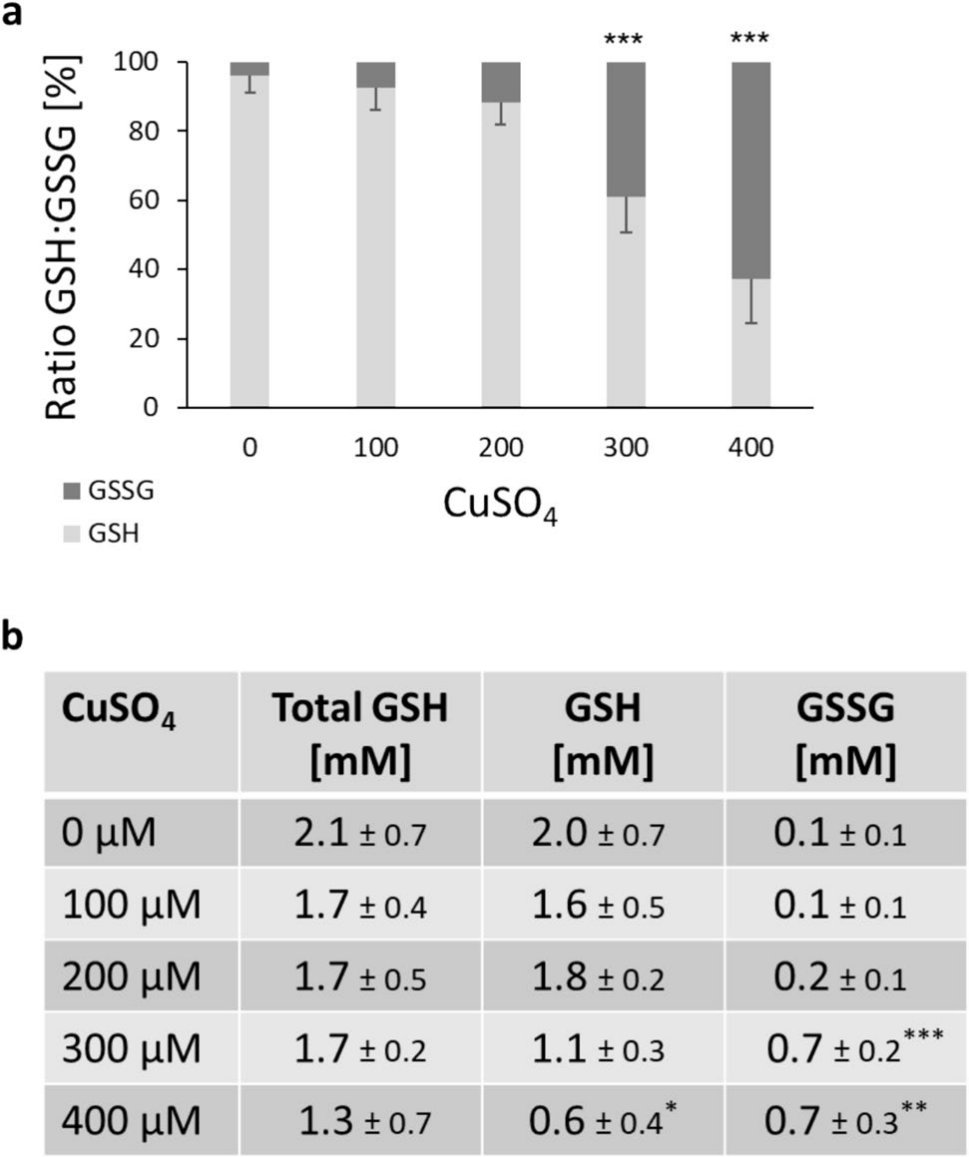
Oxidative stress: **a** Levels of glutathione (GSH) and glutathione disulfide (GSSG) were measured and effects on the ratio of GSH/GSSG [% related to total GSH] were investigated in human, differentiated neurons (LUHMES cells) following 48 h incubation with CuSO_4_. **b** Cellular levels [mM] of total GSH, GSH, and GSSG were calculated. Shown are mean values of at least three independent experiments ± SD. * *p* < 0.05, ** *p* < 0.01, *** *p* < 0.001 (compared to untreated control) based on ANOVA one-way test followed by Dunnett’s test

To assess the antioxidative capacity of the neurons in response to Cu treatment, the GSH homeostasis was investigated as marker for oxidative stress. GSH homeostasis was already disrupted at 300 µM Cu with a GSH/GSSG ratio of about 60%/40% (see Fig. 3a). It seems that the antioxidant defense mechanisms are activated already at subcytotoxic Cu levels. Interestingly, in contrast to previous studies with human astrocytes, total GSH levels decreased with increasing Cu incubation, while oxidized GSSG rose (see Fig. 3b), providing evidence for reduced antioxidative capacity in the neurons at higher Cu levels (Witt et al. 2021).

### Neuronal network

**Fig. 4:**
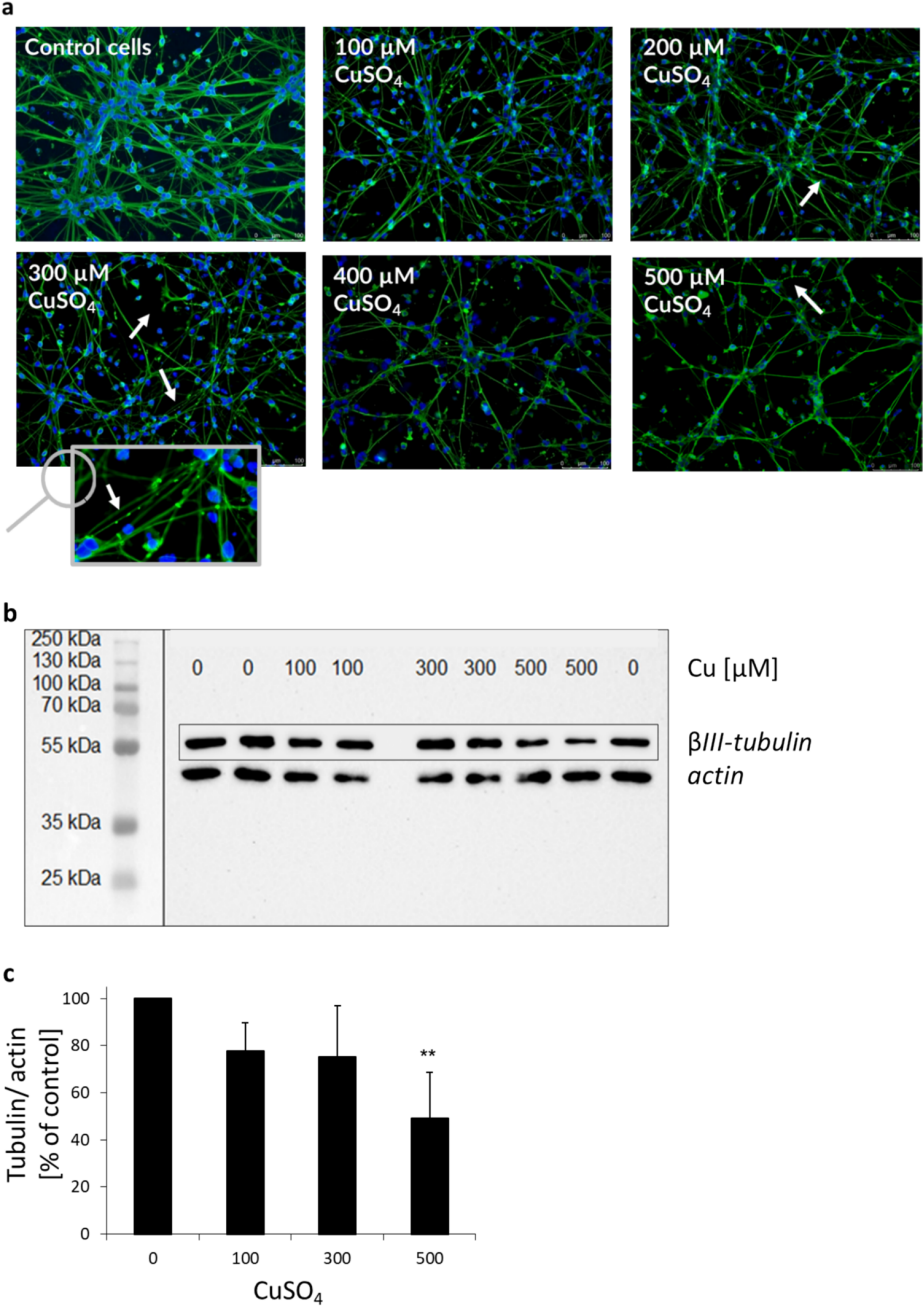
Neuronal network: **a** βIII-tubulin (green) and cell nuclei (blue) were stained in human, differentiated neurons (LUHMES cells) in non-treated control cells or following 48 h incubation with CuSO_4_. Shown are exemplary images of the analyzed slides using 20 × objective. Magnifying glass indicates zoomed image of 300 µM CuSO_4_ showing dendritic bead formations. **b** βIII-tubulin protein levels were investigated via western blot analysis in human, differentiated neurons (LUHMES cells) following 48 h incubation with CuSO_4_. Actin was used as reference protein. **c** Effects on the protein expression levels were analyzed in human, differentiated neurons (LUHMES cells) following 48 h incubation with CuSO_4_. Shown are mean values of at least three independent experiments + SD. ** *p* < 0.01 (compared to untreated control) based on ANOVA one-way test followed by Dunnett’s test (color figure online)

Effects on the neuronal network were assessed via immunostaining of βIII-tubulin. Neurite toxicity turned out to be a highly sensitive endpoint in context of Cu toxicity. Cu showed pronounced effects on the integrity of the neuronal network, already at subcytotoxic levels of 300 µM Cu (see Fig. 4a). At 500 µM Cu, the neuronal network was massively disrupted. Interestingly, accumulation processes, so-called dendritic bead formations, were observed at the damaged neurites (see Fig. 4a zoom).

Western blot technique was used to study effects on the cellular expression of βIII-tubulin on protein level. The expression of this cytoskeletal protein decreased with increasing Cu levels (see Fig. 4b). To account for protein expression levels, band intensities of βIII-tubulin were related to the reference protein actin. A moderate decrease in βIII-tubulin expression was already observed at subcytotoxic levels of 100 µM Cu (see Fig. 4c). 500 µM Cu induced a massive reduction of the cytoskeletal protein.

### Cellular copper levels and element homeostasis

**Fig. 5:**
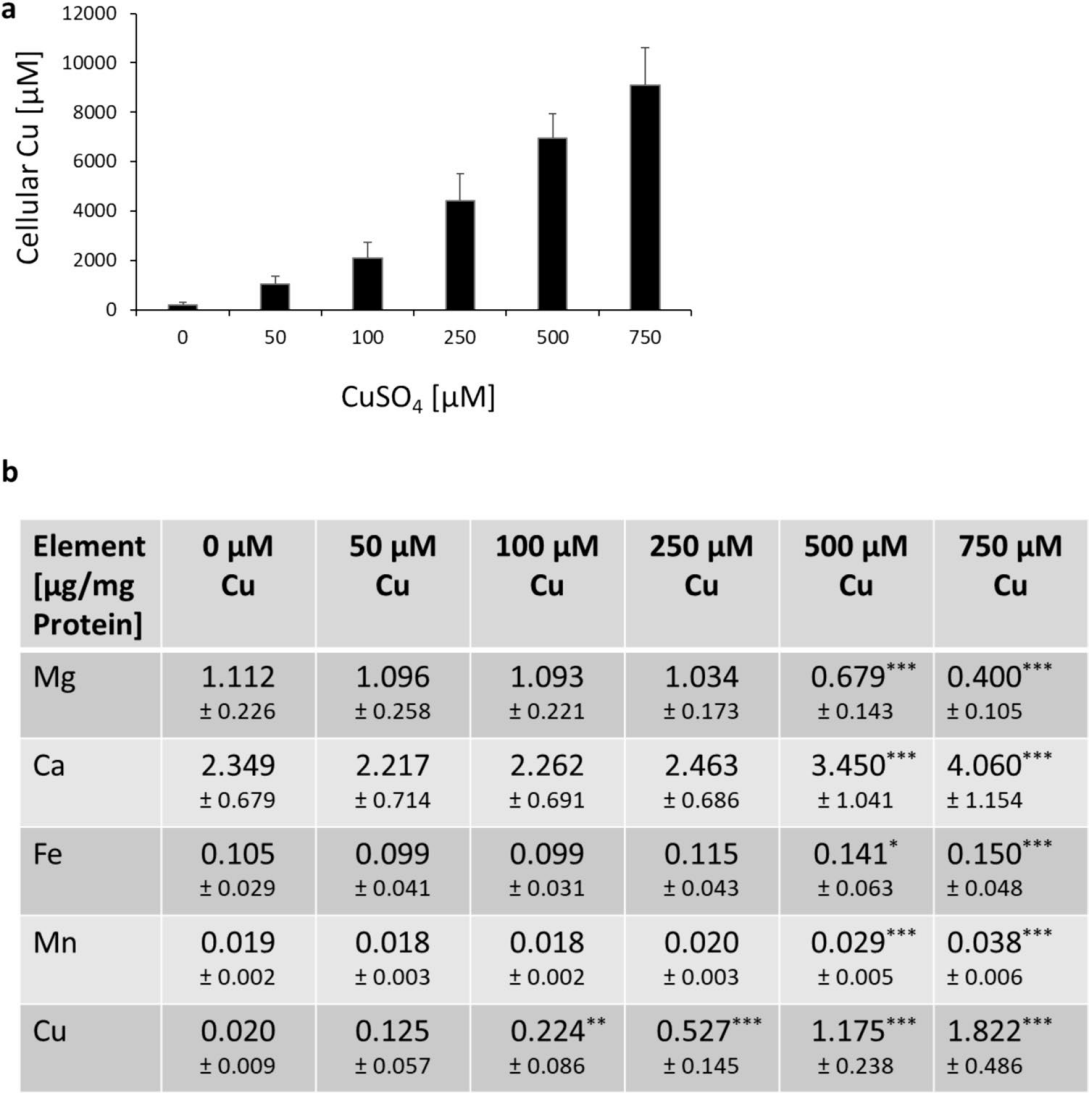
Cellular element homeostasis: **a** Cellular Cu levels were assessed via ICP-MS/MS in cell lysates of human, differentiated neurons (LUHMES cells) following 48 h incubation with CuSO_4_. **b** Cellular element levels of Mg, Ca, Fe, Mn, and Cu were investigated via ICP-MS/MS in cell lysates of human, differentiated neurons (LUHMES cells) following 48 h incubation with CuSO_4_. Shown are mean values of at least three independent experiments ± SD. * *p* < 0.05, ** *p* < 0.01, *** *p* < 0.001 (compared to untreated control) based on ANOVA one-way test followed by Dunnett’s test

Cellular Cu levels were studied in human neurons applying ICP-MS/MS. Basal Cu levels of neurons were found to be 0.02 µg Cu/mg protein. Cellular Cu levels increased concentration-dependently with higher Cu incubation (see Fig. 5a). Accumulation factors were between 12- and 21-fold. Interestingly, when comparing with other human brain cells, astrocytes seem to accumulate less Cu as compared to the here applied neurons (Witt et al. 2021).

In parallel, the homeostases of other essential elements were analyzed to investigate influences by Cu and potential interactions among the elements. A steady decrease was observed for Mg with increasing Cu incubation. Interestingly, Ca, Fe, and Mn levels increased with rising Cu levels (see Fig. 5b).

## Discussion

This study aimed to provide mechanistic insights in terms of copper neurotoxicity using a neuronal, dopaminergic cell model. First studies on general toxicity pointed out that lysosomal integrity is highly affected by excess copper levels in the human neurons. In context of Cu toxicity, earlier in vitro studies (HepG2) also observed lysosomal disruptions assuming that lysosomal function might be disrupted by alterations in lysosomal membrane integrity such as lipid peroxidation caused by Cu-induced oxidative stress (Myers et al. 1993). However, our study rather pointed out that impaired mitochondrial activity is associated with the observed lysosomal dysfunction, since both organelles are affected at similar Cu levels in the neurons, while ROS induction seems to be a later consequence. In fact, it has been discussed that excess Cu induces lysosomal exocytosis, thereby releasing Cu from the cells and counteracting Cu dyshomeostasis (Polishchuk and Polishchuk 2016). Therefore, lysosomes seem to be involved in the neuronal defense strategy against high Cu levels.

Mitochondria, in particular brain mitochondria, are major and early targets of Cu toxicity (Borchard et al. 2018). Cu overload has been shown to destroy mitochondrial structures leading to mitochondrial impairments or even dysfunction (Borchard et al. 2018; Zischka et al. 2011). Our study substantiates these observations. Cu had pronounced effects on the mitochondrial activity, as the mitochondrial membrane potential was massively reduced in the Cu-exposed neurons. Reduced mitochondrial activity is very likely associated with impaired ATP production in connection with respiratory chain defects, finally resulting in a disrupted cellular energy homeostasis. Previous studies indicated that Cu affects complexes I, III, and IV of the respiratory chain in rat liver mitochondria and reduces ATP production in neuroblastoma cells (Borchard et al. 2018; Hosseini et al. 2014; Murphy 2009). Moreover, particularly, the impairment of complex I and IV was shown to increase Cu-induced ROS induction (Hosseini et al. 2014).

In fact, earlier studies in SH-SY5Y indicate that the generation of mitochondrial ROS is not the primary mode of action, but rather a later consequence of Cu neurotoxicity (Borchard et al. 2018). Our study also provided evidence that mitochondrial ROS are only induced at higher, cytotoxic Cu levels, substantiating that mitochondrial ROS induction is not the cause, but a later event of Cu toxicity in the neurons. The induction of oxidative stress via ROS generation is closely linked to functional impairments of mitochondria. Increased leakage of electrons associated with disrupted respiratory chain further induces oxidative stress and exacerbate the damage to mitochondria (Huang et al. 2024; Tassone et al. 2021). Further consequences are disruption of mitochondrial membrane integrity associated with lipid peroxidation and protein modifications finally leading to opening of the mitochondrial permeability transition pore, swelling of mitochondria, and induction of apoptotic cell death by cytochrome c release (Joshi et al. 2021). Our study provides first hints for an opening of the mitochondrial permeability transition pore. High levels of Ca^2+^ and low levels of Mg^2+^, as observed in the Cu-exposed neurons, are discussed as activator for this event (Bernardi et al. 2023). This, however, seems to be a later event in the course of Cu toxicity in the neurons. Further studies on cell death mechanisms are needed in the neurons to check if apoptosis is induced at excess Cu levels.

GSH is discussed to be involved in the primary cellular defense mechanisms against Cu-induced toxicity. Besides its antioxidant effects, GSH plays an important role as Cu-binding protein, protecting cells from the toxicity of free, redox active Cu (Scheiber and Dringen 2011; Scheiber et al. 2014). We observed a massively disrupted GSH homeostasis combined with reduced GSH levels in the Cu-exposed human neurons which was even more pronounced as compared to other brain cells (Witt et al. 2021). The early disrupted GSH/GSSG ratio, as marker for oxidative stress, indicates that the neurons aim to counteract against Cu-induced oxidative stress already at 300 µM Cu. Since the GSH levels are significantly reduced only at higher Cu levels, neurons seem to be able to cope with Cu-induced ROS due to the scavenging and Cu-binding activity of GSH, affirmed by the late ROS induction observed in our study. The depletion of GSH at higher Cu levels might additionally aggravate the effects of Cu toxicity. It has been shown before that GSH supplementation could prevent phospholipid peroxidation and mitochondrial dysfunction following Cu exposure in rat liver mitochondria, pointing out the pivotal role of GSH in the course of Cu toxicity (Saporito-Magrina et al. 2018).

In our study, the neuronal network was massively disrupted following Cu exposure. These findings are supported by western blot analysis of the cytoskeletal protein, indicating cytoskeletal degradation in the neurons. Interestingly, characteristic accumulation processes, so-called bead formations, were observed at the damaged neurites. Microtubule destabilization and neuritic beading are likely caused by impaired mitochondrial function, which was also seen in our study (Cho et al. 2021). In fact, mitochondrial dysfunction coincident with neuritic beading is discussed as early hallmark of neuronal toxicity likely resulting in impaired neuronal signaling associated with pathological conditions (Greenwood et al. 2007). In this context, consequences of oxidative stress induced by Cu might play an important role, leading to peroxidation of membrane lipids, disruption of the physiological function, and signal transmission. Increased lipid peroxidation due to Cu overload has been observed in neurons of a Wilson disease mice model, before (White et al. 1999). In addition, Wilson disease patients showed elevated levels of lipid peroxidation along with diminished GHS levels, providing evidence for reduced antioxidative capacity as well (Kalita et al. 2014). Future studies should address the question whether and how these adverse conditions affect the neuronal signaling.

Interestingly, excess Cu levels not only disrupted the Cu homeostasis in the human neurons, but also affected the element homeostasis of Mg, Ca, Mn, and Fe. Mg plays a pivotal role in the brain, including ion channel function, mitochondrial membrane stability/oxidative phosphorylation, and cellular signaling. Imbalances have been associated with cellular stress and disrupted energy metabolism (Bernardi et al. 2023; Welch and Ramadan 1995; Yamanaka et al. 2019). Reduced Mg levels might contribute to the Cu-induced mitochondrial disruption in this study as discussed before. Depletion of Mg could be also observed in brain samples of patients suffering from Cu-associated diseases, where Cu was markedly accumulated as well (Faa et al. 2001; Wang and Wang 2017; Yamanaka et al. 2019). Ca is an important second messenger involved in cell death mechanisms. Elevated cellular Ca levels might be a sign for Cu-induced apoptosis triggered in the neurons. An increase in Ca levels has been reported in Cu-treated neurons, before, and this could be clearly linked to Cu-induced apoptosis (Chen et al. 2009). In fact, elevated intracellular Ca levels have been also observed in context of Alzheimer’s disease (Everett et al. 2018). A cellular increase in the redox active Fe and Mn is likely to be associated with the induction of oxidative stress (Tarnacka et al. 2021). Consequently, imbalances in these elements might contribute to Cu-induced neurotoxicity and might promote the progression of neurodegenerative processes. In fact, it is discussed that high Mn levels affect the expression of Cu exporters including ATP7A and ATP7B, further enhancing cellular Cu accumulation (Fu et al. 2014). Elevated Fe and Mn levels have been reported in brains of patients suffering from Cu-associated diseases, before (Cicero et al. 2017; Roberts et al. 2012).

Causes for the disturbed homeostasis are not completely understood. Further studies are needed to elucidate interplays among the different elements and their roles in the neurotoxic mechanisms in context of neurological diseases. Moreover, it is essential to study the subcellular element distribution with high-resolution bioimaging techniques. Based on these techniques, local shifts such as accumulation of certain elements in organelles or co-accumulations in plaques can be detected (Witt et al. 2020). Future studies in this context will shed light on the molecular and subcellular mechanisms responsible for the onset and progression of Cu-mediated neurodegenerative diseases.

## Summary and conclusion

The objective of this study was to characterize the neurotoxic mechanisms of Cu in human, dopaminergic neurons (LUHMES cells). Pronounced cytotoxic effects with focus on lysosomal integrity were observed at 420 µM (EC_30_) following 48 h of incubation. In fact, mitochondrial function and neuronal network were mostly affected by Cu (significantly already at 100 µM Cu). In this context, it seems that Cu affects both the expression and subcellular distribution of βIII-tubulin in the human neurons. Moreover, the GSH homeostasis was massively disrupted at 300 µM Cu. Besides playing an important role in antioxidative defense, GSH is also involved in Cu homeostasis as Cu-binding protein. Following Cu exposure, total GSH levels decreased in the neurons, indicating both reduced antioxidative and Cu-binding capacity. However, ROS generation seems to be rather a later consequence of Cu toxicity, since ROS were only induced at higher Cu levels. A concentration-dependent uptake of Cu was observed in the neurons. Interestingly, Cu turned out to interfere with the homeostasis of other essential elements. Particularly altered cellular levels of Mg, Ca, and Fe were observed with increasing Cu incubation. Altered Fe and Mn homeostases likely exacerbate adverse effects of a Cu overload by inducing oxidative stress in the cells. Therefore, these elements might contribute to neurotoxic mechanisms induced by Cu imbalances. Their exact roles and interplays in the course of Cu neurotoxicity need to be characterized in future studies.

Studies on subcellular levels using high-resolution bioimaging techniques are urgently needed, to detect local shifts in cellular element distributions (e.g., accumulation in organelles or plaques). In addition, mitochondrial fraction might be used to specifically characterize mitochondrial impairments induced by Cu focusing on the respiratory chain and element profiles. Findings in this context will contribute to the understanding of pathological conditions associated with Cu dyshomeostasis and help to derive suitable biomarkers and to develop approaches for therapeutic strategies against Cu-related neurodegenerative diseases.

## Data Availability

The authors declare that the data supporting the findings of this study are available within the paper. Should any raw data files be needed in another format they are available from the corresponding author upon reasonable request.

## Abbreviations

DMEM: Dulbecco’s modified Eagle’s medium
EC_30_: Effective concentration with 30% impact
GSH: Glutathione
GSSG: Glutathione disulfide
LUHMES: Lund human mesencephalic
ROS: Reactive oxygen species
RT: Room temperature
